# Hemoglobin and Perihematomal Edema After Intracerebral Hemorrhage: A Post Hoc Analysis of the i-DEF Trial

**DOI:** 10.1007/s12028-025-02284-3

**Published:** 2025-05-21

**Authors:** Alexandros A. Polymeris, Vasileios-Arsenios Lioutas, Sarah Marchina, David J. Seiffge, David J. Roh, Fernanda Carvalho Poyraz, Magdy H. Selim

**Affiliations:** 1 Stroke Division, Department of Neurology, Beth Israel Deaconess Medical Center and Harvard Medical School, Boston, MA USA; 2 Department of Neurology and Stroke Center, University Hospital Basel and University of Basel, Basel, Switzerland; 3 Department of Neurology, Inselspital University Hospital Bern and University of Bern, Bern, Switzerland; 4 Department of Neurology, Vagelos College of Physicians and Surgeons, Columbia University, New York City, NY USA; 5 Department of Neurosurgery, Icahn School of Medicine at Mount Sinai, New York City, NY USA

**Keywords:** Intracerebral hemorrhage, Anemia, Hemoglobin, Perihematomal edema, Edema extension distance

## Abstract

**Background:**

Anemia is common after intracerebral hemorrhage (ICH). It has been attributed to inflammation and is associated with poor outcomes. We investigated whether this could be related to the effects of hemoglobin (Hb) on perihematomal edema (PHE).

**Methods:**

We performed an exploratory post hoc analysis of the Intracerebral Hemorrhage Deferoxamine (i-DEF) randomized controlled trial. We included participants with primary supratentorial ICH, available baseline Hb levels, and computed tomography scans at baseline and follow-up after 72–96 h. We investigated the association of Hb and anemia (as continuous and dichotomous exposures, respectively) with edema extension distance (EED) as the main continuous outcome at baseline and follow-up and as its interscan change using Spearman correlation and unadjusted and adjusted linear models. We examined absolute and relative PHE in ancillary analyses.

**Results:**

We analyzed data from 276 of 293 (94%) i-DEF participants. The median age was 61 (interquartile range [IQR] 52–70) years, and 39% of participants were female. The median Hb level was 14.1 (IQR 13–15.2) g/dL, and 41 participants (15%) were anemic. The median EED was 4.4 (IQR 3.5–5.3) mm at baseline and 6.4 (IQR 5.3–7.3) mm at follow-up. Hb was weakly inversely correlated with baseline (*ρ* =  − 0.12, *p* = 0.05) and follow-up EED (*ρ* =  − 0.11, *p* = 0.07) but not with interscan EED change (*ρ* =  − 0.01, *p* = 0.89). Linear models showed similar relationships of Hb with baseline and particularly follow-up EED but not with EED change. In ancillary analyses, absolute and relative PHE showed no clear correlation with Hb but maintained similar relationships with Hb in linear models as in the main analysis.

**Conclusions:**

We identified signals for an association of baseline Hb with PHE after ICH. These findings may warrant further exploration in larger cohorts.

**Clinical trial registration:**

ClinicalTrials.gov identifier: NCT02175225.

**Supplementary Information:**

The online version contains supplementary material available at 10.1007/s12028-025-02284-3.

## Introduction

Anemia is common after intracerebral hemorrhage (ICH) and has been linked to poor outcomes [1–3], but the underlying mechanisms are unclear. Several studies investigated the association of hemoglobin (Hb) with hematoma volume and its expansion as potential outcome mediators with mixed results [1–4]. However, hardly any data exist about the effects of Hb on the formation and evolution of perihematomal edema (PHE), which is thought to represent the radiological marker of secondary injury after ICH [5]. Anemia and PHE might share a common pathogenesis involving systemic and local inflammatory mechanisms [5, 6].

To inform future research into anemia as a potential therapeutic target, a link between Hb and PHE is worth exploring. We investigated this using high-quality, prospectively collected data from the Intracerebral Hemorrhage Deferoxamine (i-DEF) trial [7].

## Methods

### Study Design and Participants

The i-DEF (ClinicalTrials.gov identifier: NCT02175225) trial was a multicenter randomized, placebo-controlled phase 2 clinical trial conducted across 40 centers in the United States and Canada. The trial investigated the iron chelator deferoxamine mesylate as a treatment option against secondary brain injury after ICH, in which iron from hemolyzed blood has been implicated. Its methodology, including details about data collection and imaging analysis, is described elsewhere [7, 8]. In short, i-DEF participants had primary supratentorial ICH and were randomized to receive study drug infusions for three consecutive days, starting within 24 h of ICH onset. Patients with known severe anemia (Hb level < 7 g/dL or requiring blood transfusions), coagulopathy (prolonged activated partial thromboplastin time or elevated international normalized ratio on presentation, or use of direct oral anticoagulants or low-molecular-weight heparin), planned hematoma evacuation, and infratentorial or secondary ICH were excluded from participation. Screening in the i-DEF trial included standardized laboratory assessments, including hematology, done locally at each site as part of routine care. Head computed tomography (CT) was obtained at screening (baseline scan) and within 24 h of completion of the last infusion (i.e., within 72–96 h from ICH onset; follow-up scan). CT scans were sent to a core imaging laboratory (Beth Israel Deaconess Medical Center, Boston, MA). Imaging evaluations were done by experienced blinded raters using an imaging analysis software (Analyze 11.0; AnalyzeDirect, Overland Park, KS). Volumetric assessments of intracerebral hematoma and PHE were done on CT scans using a validated Hounsfield-unit-threshold-based, semiautomated segmentation approach with manual correction, as described previously and in Supplementary Fig. 1 [7, 8].

In this exploratory post hoc analysis focusing on the association of Hb with PHE, we included all i-DEF participants with available laboratory and imaging data. We excluded those with emergent surgical treatment (craniectomy/hematoma evacuation) because this may impact PHE growth.

### Exposures and Outcomes

The main exposures were Hb (as a continuous variable) and anemia (as a dichotomous one) at screening. The latter was defined as Hb level < 12 g/dL for women and < 13 g/dL for men as per standard World Health Organization definitions.

The main outcome was PHE assessed as edema extension distance (EED; as continuous variable, expressed in mm, defined as the difference between the radius of a sphere equal to the combined ICH and PHE volume and the radius of a sphere equal to the ICH volume alone) [9]. We assessed EED as a single measurement on the baseline and follow-up scans, and as its interscan change. We chose EED as the main outcome because it has been shown to be independent of hematoma volume and more advantageous over other metrics to study the effects of exposures on PHE [9, 10]. In ancillary analyses, we similarly investigated absolute PHE volume (aPHE) and relative PHE (rPHE; defined as the ratio of aPHE to ICH volume), both as continuous outcomes. Although only rPHE was investigated in the main i-DEF trial, subsequent analyses have reported on aPHE and EED in the i-DEF data set before [7, 8].

### Statistical Analysis

We present all data using descriptive statistics. We compared categorical and continuous variables using the *χ*^2^ test or Fisher’s exact test and the Mann–Whitney *U*-test or *t*-test, respectively, as appropriate. We assessed the relationship between Hb and PHE measures using Spearman correlation. We further investigated the association of Hb or anemia with PHE measures in (1) unadjusted, (2) partially adjusted, and (3) fully adjusted linear regression models. Partially adjusted models included as covariates known determinants of Hb (sex, age, and race) [11] and the most essential potential determinants of baseline PHE [12] (baseline ICH volume and time from ICH onset to baseline scan) or its progression (interscan time, use of hyperosmolar therapy [hypertonic saline and/or mannitol], and actual i-DEF treatment received [deferoxamine or placebo]). Fully adjusted models additionally included the following potential outcome modifiers: serum glucose level, ICH location (lobar, thalamic, or deep nonthalamic), ICH score (comprising Glasgow Coma Scale score, age, ICH volume, intraventricular hemorrhage, and infratentorial ICH location), previous use of antithrombotics (antiplatelets or anticoagulants), and external ventricular drain placement before follow-up scan. We chose to include external ventricular drain placement because this may capture several relevant aspects worth accounting for, including its potential treatment effects and a broader reflection of morbidity and ICH severity [13, 14]. There were no missing values in any of these covariates. Because of skewed distributions, we log-transformed ICH volume, aPHE, and rPHE for regression analyses. We report correlation coefficients (*ρ*) and model-based (β coefficient) estimates with 95% confidence intervals (CIs) as measures of association. For log-transformed PHE measures, we report back-transformed estimates (*β*_mult_), which represent multiplicative effects on the geometric mean of the PHE measure. We present *p* values for all tests but refrain from defining significance thresholds or from correcting for multiple testing given the exploratory nature of the study. We performed all analyses using STATA 18.0 (StataCorp LLC, College Station, TX). We conducted this study in accordance with the Strengthening the Reporting of Observational Studies in Epidemiology statement.

## Results

Of 293 i-DEF participants, 1 lacked baseline imaging evaluation, 8 underwent emergent surgical treatment, and 8 lacked follow-up imaging assessment, leaving 276 participants (median age 61 years, 39% female) available for analysis. Information on Hb was complete. At baseline, the median Hb level was 14.1 g/dL (women 13.1 g/dL, men 14.5 g/dL), and 41 participants (15%) were anemic. The median ICH and aPHE volumes were 12.4 (interquartile range [IQR] 6.3–23.6) mL and 14.8 (IQR 8.4–24.1) mL, respectively. The median EED was 4.4 (IQR 3.5–5.3) mm. Compared to nonanemic participants, anemic ones tended to have greater EED both at baseline (4.8 [IQR 4.1–5.5] mm vs. 4.4 [IQR 3.4–5.2] mm, *p* = 0.05) and follow-up (6.9 [IQR 6.0–7.8] mm vs. 6.3 [IQR 5.3–7.3] mm, *p* = 0.05). All remaining PHE measures showed numerically higher values in anemic participants, but between-group differences were less pronounced (median aPHE 16.3 [IQR 9.2–25.3] mL vs. 14.2 [IQR 8.4–23.4] mL, *p* = 0.28 at baseline and 24.0 [IQR 16.3–46.8] mL vs. 22.3 [IQR 14.6–38.4] mL, *p* = 0.37 at follow-up; median rPHE 1.33 [IQR 1.07–1.82] vs. 1.15 [IQR 0.95–1.53], *p* = 0.07 at baseline and 2.25 [IQR 1.74–3.00] vs. 1.94 [IQR 1.55–2.66], *p* = 0.11 at follow-up). All clinical, laboratory, and radiological data stratified to presence or absence of anemia are summarized in Table 1.

**Table 1:** Clinical, laboratory, and imaging data

	Total (*N* = 276)	No anemia (*n* = 235)	Anemia (*n* = 41)	*p* value
Age, years, median (IQR)	61 (52–70)	60 (52–69)	63 (55–71)	0.12
Female sex, *n* (%)	107 (39)	86 (37)	21 (51)	0.08
Race, *n* (%)				
White	170 (61.6)	148 (63.0)	22 (53.7)	0.64
Black	61 (22.1)	49 (20.9)	12 (29.3)	
Asian	37 (13.4)	31 (13.2)	6 (14.6)	
Other	8 (2.9)	7 (3.0)	1 (2.4)	
*Medical history,* *n* (%)				
Hypertension	227 (82)	195 (83)	32 (78)	0.45
Diabetes mellitus	75 (27)	62 (26)	13 (32)	0.48
Cardiac disease	27 (10)	21 (9)	6 (15)	0.26
Previous ischemic stroke	26 (9)	24 (10)	2 (5)	0.28
Previous ICH	10 (4)	10 (4)	0 (0)	0.18
Antiplatelet use	89 (32.2)	75 (31.9)	14 (34.1)	0.78
Anticoagulant use	4 (1.4)	4 (1.7)	0 (0)	0.40
*Clinical, radiological, and laboratory data at baseline*				
GCS score, median (IQR)	14 (12–15)	14 (12–15)	13 (12–15)	0.55
NIHSS score, median (IQR)	13 (8–17)	12 (8–17)	14 (8–17)	0.76
ICH onset to baseline CT scan, median (IQR), h	3.3 (1.3–7.0)	3.3 (1.3–6.7)	3.3 (1.6–8.4)	0.66
ICH location, *n* (%)				
Lobar	49 (18)	41 (17)	8 (20)	0.86
Thalamic	102 (37)	86 (37)	16 (39)	
Deep nonthalamic	125 (45)	108 (46)	17 (42)	
ICH volume, median (IQR), mL	12.4 (6.3–23.6)	12.5 (6.4–23.0)	11.9 (6.2–27.4)	0.91
IVH, *n* (%)	114 (41.3)	97 (41.3)	17 (41.5)	0.98
IVH volume, median (IQR), mL	5.8 (1.7–14.2)	5.7 (1.8–14.2)	6.2 (0.4–12.2)	0.41
ICH score, median (IQR)	1 (0–1)	1 (0–1)	1 (0–1)	0.38
EED, median (IQR), mm	4.4 (3.5–5.3)	4.4 (3.4–5.2)	4.8 (4.1–5.5)	0.05
aPHE, median (IQR), mL	14.8 (8.4–24.1)	14.2 (8.4–23.4)	16.3 (9.2–25.3)	0.28
rPHE, median (IQR)	1.18 (0.97–1.56)	1.15 (0.95–1.53)	1.33 (1.07–1.82)	0.07
Hb, median (IQR), g/dL	14.1 (13.0–15.2)	14.3 (13.5–15.3)	11.4 (11–12.2)	< 0.001
Serum glucose, median (IQR), mg/dL	137 (115.5–162)	135 (114–162)	146 (123–163)	0.28
*Treatment details,* *n* (%)				
Antiedema agents	37 (13)	30 (12.8)	7 (17.1)	0.46
Deferoxamine	142 (51)	120 (51.1)	22 (53.7)	0.76
EVD placement	31 (11)	24 (10.2)	7 (17.1)	0.20
*Radiological data at follow-up*				
ICH onset to follow-up CT scan, median (IQR), h	89.0 (81.5–95.0)	89.0 (81.2–94.9)	89.2 (82.1–95.5)	0.84
Baseline to follow-up CT scan, median (IQR), h	82.6 (77.2–90.3)	82.6 (77.2–90.2)	82.4 (76.9–90.8)	0.88
ICH volume, median (IQR), mL	11.8 (6.3–23.7)	12.0 (6.3–23.7)	10.9 (6.0–25.1)	0.95
EED, median (IQR), mm	6.4 (5.3–7.3)	6.3 (5.3–7.3)	6.9 (6.0–7.8)	0.05
Interscan change, median (IQR), mm	2.0 (1.1–2.8)	1.9 (1.1–2.8)	2.4 (1.3 – 2.9)	0.46
aPHE, median (IQR), mL	22.6 (14.9–38.4)	22.3 (14.6–38.4)	24.0 (16.3–46.8)	0.37
Interscan change, median (IQR), mL	8.1 (4.1–15.1)	7.9 (4.0–15.0)	8.6 (5.4–16.5)	0.36
rPHE, median (IQR)	2.02 (1.56–2.74)	1.94 (1.55–2.66)	2.25 (1.74–3.00)	0.11
Interscan change, median (IQR)	0.79 (0.39–1.24)	0.79 (0.39–1.22)	0.85 (0.38–1.43)	0.64

Anemia was defined as Hb level < 12 g/dL (women) or < 13 g/dL (men)

aPHE, absolute perihematomal edema volume, CT, computed tomography, EED, edema extension distance, EVD, external ventricular drain, GCS, Glasgow Coma Scale, Hb, hemoglobin, ICH, intracerebral hemorrhage, IQR, interquartile range, IVH, intraventricular hemorrhage, NIHSS, National Institutes of Health Stroke Scale, rPHE, relative perihematomal edema

**Fig. 1:**
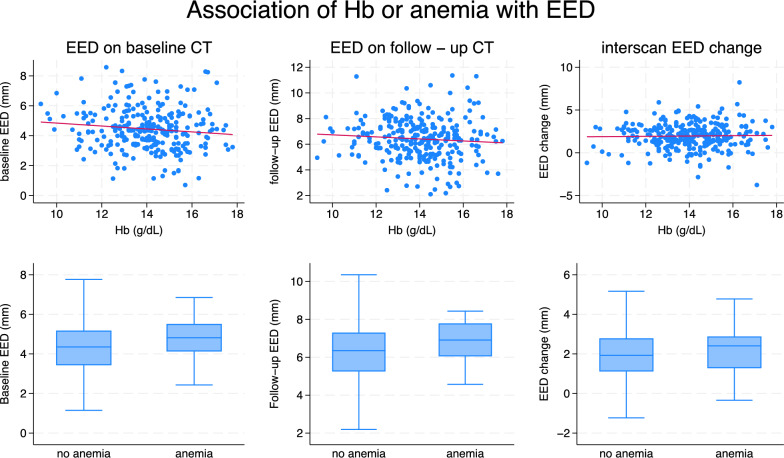
Association of Hb or anemia with EED at baseline and follow-up and its interscan change. In the scatter plots (upper panel), the red line represents the predicted values from the unadjusted linear models. In the box plots (lower panel), boxes show the median and IQR, and the whiskers show the full range of values, excluding outliers more than 1.5 times the IQR beyond the IQR limits. CT computed tomography, EED edema extension distance, Hb hemoglobin, IQR interquartile range

In our main analysis of EED, Hb was weakly inversely correlated with baseline (*ρ* =  − 0.12, *p* = 0.05) and follow-up EED (*ρ* =  − 0.11, *p* = 0.07) but not with its interscan change (*ρ* =  − 0.01, *p* = 0.89). Using linear models with and without covariate adjustments, we identified similar relationships of Hb as a continuous exposure, particularly with follow-up EED. Assessing anemia as a dichotomous exposure yielded consistent results. We did not identify relationships of our exposures with interscan EED change in any of the linear models. Detailed results are given in Table 2 and Fig. 1.

**Table 2:** Model-based estimates for the association of Hb and anemia with PHE measures at baseline, follow-up, and their change

	PHE measure at baseline			PHE measure at follow-up			Interscan change in PHE measure		
	Unadjusted	Partially adjusted^a^	Fully adjusted^b^	Unadjusted	Partially adjusted^c^	Fully adjusted^d^	Unadjusted	Partially adjusted^c^	fully adjusted^d^
EED									
Hb									
*β* (95% CI)	− 0.10 (− 0.20–0.01)	− 0.07 (− 0.17–0.04)	− 0.08 (− 0.18–0.02)	− 0.08 (− 0.20–0.04)	− 0.10 (− 0.22–0.02)	− 0.11 (− 0.23–0.00)	0.02 (− 0.09–0.13)	− 0.04 (− 0.16–0.08)	− 0.02 (− 0.14–0.10)
*p* value	0.06	0.21	0.13	0.19	0.10	0.06	0.73	0.55	0.73
Anemia									
*β* (95% CI)	0.41 (− 0.05–0.88)	0.32 (− 0.11–0.75)	0.32 (− 0.11–0.75)	0.45 (− 0.09–0.99)	0.48 (− 0.01–0.97)	0.51 (0.03–0.98)	0.04 (− 0.40–0.54)	0.16 (− 0.33–0.65)	0.13 (− 0.35–0.62)
*p* value	0.08	0.14	0.14	0.10	0.05	0.04	0.88	0.53	0.58
aPHE									
Hb									
*β*_mult_ (95% CI)	0.96 (0.90–1.02)	0.98 (0.94–1.01)	0.97 (0.94–1.01)	0.97 (0.92–1.02)	0.97 (0.94–1.00)	0.97 (0.94–1.00)	1.00 (0.98–1.03)	0.99 (0.97–1.02)	1.00 (0.97–1.03)
*p* value	0.17	0.17	0.10	0.23	0.06	0.04	0.90	0.61	0.87
Anemia									
*β*_mult_ (95% CI)	1.17 (0.88–1.55)	1.10 (0.96–1.27)	1.10 (0.96–1.26)	1.15 (0.90–1.47)	1.13 (0.99–1.29)	1.14 (1.00–1.29)	1.04 (0.93–1.17)	1.05 (0.94–1.18)	1.05 (0.94–1.18)
*p* value	0.29	0.16	0.16	0.28	0.07	0.05	0.52	0.39	0.40
rPHE									
Hb									
*β*_mult_ (95% CI)	0.98 (0.94–1.01)	0.98 (0.94–1.01)	0.97 (0.94–1.01)	0.99 (0.95–1.02)	0.98 (0.95–1.01)	0.97 (0.94–1.00)	1.00 (0.98–1.01)	0.99 (0.98–1.00)	0.99 (0.98–1.00)
*p* value	0.16	0.17	0.10	0.37	0.14	0.05	0.39	0.15	0.15
Anemia									
*β*_mult_ (95% CI)	1.11 (0.94–1.31)	1.10 (0.96–1.27)	1.10 (0.96–1.26)	1.10 (0.95–1.28)	1.12 (0.99–1.27)	1.13 (1.01–1.27)	1.02 (0.97–1.07)	1.03 (0.97–1.08)	1.03 (0.97–1.08)
*p* value	0.21	0.16	0.16	0.21	0.06	0.04	0.50	0.33	0.34

*β*_mult_ denotes multiplicative effects, so that *β*_mult_ > 1 indicates a positive association, and *β*_mult_ < 1 indicates a negative one

aPHE, absolute perihematomal edema volume, CI, confidence interval, EED, edema extension distance, Hb, hemoglobin, ICH, intracerebral hemorrhage, i-DEF, Intracerebral Hemorrhage Deferoxamine, PHE, perihematomal edema, rPHE, relative perihematomal edema

^a^Adjusted for sex, age, race, baseline ICH volume (log-transformed), and time from ICH onset to baseline scan

^b^Adjusted for sex, age, race, baseline ICH volume (log-transformed), time from ICH onset to baseline scan, serum glucose level, ICH score, ICH location, and use of antithrombotics

^c^Adjusted for sex, age, race, baseline ICH volume (log-transformed), time from ICH onset to baseline scan, antiedema treatment, i-DEF treatment, and interscan time

^d^Adjusted for sex, age, race, baseline ICH volume (log-transformed), time from ICH onset to baseline scan, antiedema treatment, i-DEF treatment, interscan time, serum glucose level, ICH score, ICH location, use of antithrombotics, and EVD placement

In ancillary analyses, there was no clear correlation between Hb and aPHE (baseline *ρ* =  − 0.09, *p* = 0.14; follow-up *ρ* =  − 0.09, *p* = 0.15; interscan change *ρ* =  − 0.03, *p* = 0.59) or rPHE (baseline *ρ* =  − 0.08, *p* = 0.18; follow-up *ρ* =  − 0.05, *p* = 0.36; interscan change *ρ* =  − 0.01, *p* = 0.92). In linear models, there were signals for an association of our exposures with aPHE and rPHE at follow-up and less so at baseline but not with their interscan change (Table 2).

## Discussion

This exploratory post hoc analysis of participants with primary small to moderately sized supratentorial ICH from the i-DEF trial showed weak signals for an association of Hb with PHE, particularly as assessed by means of EED.

In patients with ICH, both anemia [1, 2, 6] and PHE [15] are considered predictors of clinical outcome, with anemia increasingly discussed as a promising therapeutic target [3, 16] and PHE as the radiological surrogate for secondary injury [5]. Importantly, both are thought to arise, at least in part, from inflammatory mechanisms. In particular, systemic inflammation seemed to drive the evolution of anemia in a recent study using iron biomarker profiling and systemic inflammatory response syndrome scores [6]. Markers of systemic inflammation, including the neutrophil/lymphocyte ratio and the C-reactive protein level, have also been associated with PHE formation and evolution in some [17, 18] but not all studies [19, 20]. Given these associations, it is intriguing that the link between Hb and PHE seemed most pronounced when assessing PHE by means of EED rather than the actual PHE volume in our study. Being largely independent of hematoma volume, EED is thought to reflect the perihematomal inflammatory response more accurately than other PHE measures and is known to require smaller sample sizes to detect potential effects of exposures on PHE [9, 10]. Considering this, our findings might be indicative of a true underlying association between Hb and PHE, which our sample may have been underpowered to fully uncover.

Notably, Hb showed signals for association only with PHE assessed as a single measurement, particularly at follow-up. However, the evolution of PHE over the first 3–4 days after ICH appeared largely unaffected by baseline Hb level, regardless of the PHE measure analyzed. This suggests that correcting anemia at baseline might not impact the trajectory of PHE, which in turn has been shown to bear the largest prognostic significance [15]. Taken together, our findings offer little support to the hypothesis that any outcome-modifying effects of anemia might be mediated to a substantial degree via its effects on PHE but yielded signals that preclude definitive conclusions and merit further investigation in larger cohorts.

Strengths of our study include (1) its high quality of data, obtained prospectively in a standardized manner within a randomized trial, with low missingness and central imaging assessments, and (2) the use of several PHE measures and assessment time points, as well as extensive adjustment for confounding, which limits the risk of spurious findings. We acknowledge the following limitations: (1) Because only a single baseline Hb measurement was available, we cannot exclude that subsequent changes to Hb levels might have yielded different results, given that the prevalence of anemia increases within the first days of ICH onset, and dynamic Hb decrements have been shown to be independently associated with relevant radiological and clinical outcomes [6, 21]. This might explain the apparent lack of association of baseline Hb level with the evolution of PHE in our study; whether the development of anemia at follow-up rather than a static baseline Hb measurement is more pertinent to the evolution of PHE remains to be determined and requires further study. (2) Despite our sizable sample of more than 250 participants, our findings may be attributable to limited power in a selected trial population of less severely ill patients. Indeed, the i-DEF trial excluded patients with severe anemia, and only 15% of participants were anemic in our data set. Moreover, most i-DEF participants had small to moderately sized ICH. Given that PHE is known to be related to hematoma size [5], this may have led to less pronounced PHE responses and limited our ability to detect its association with Hb in the i-DEF trial. The infrequent use of antiedema agents at only 17% in the i-DEF trial may further support this notion. (3) Our study lacks data on iron biomarkers, which may be involved in processes driving PHE [22, 23] and might thus underlie any of its associations with Hb. Furthermore, we were unable to adjust our analyses for hematocrit because this was not collected as part of the trial. Hematocrit might influence the Hounsfield unit of the hematoma (and might interfere with Hounsfield-unit-threshold-based volumetric assessments) [24, 25] and has been shown to impact the timing of peak PHE formation [26]. However, this likely had little impact on our findings because we used a broad threshold of Hounsfield units for our automated assessment, coupled with manual correction, and did not evaluate peak PHE as one of our PHE parameters. (4) Although adjusted for several aspects of treatment, our analyses did not account for intraventricular fibrinolysis, which was not collected in the i-DEF trial but might have introduced confounding, or for the use of red blood cell transfusions during follow-up. Similarly, platelet transfusions may influence PHE [27] but were also unavailable for analysis because they were not collected. However, a standardized trial-specific ICH management protocol cautioned against their routine use at all participating sites, in accordance with guideline recommendations. (5) Our findings may not apply to patients with ICH related to anticoagulation, which may affect PHE measures [28], because such patients were largely excluded from participation in the i-DEF trial. (6) The timing of the follow-up at a median of 89 h after ICH onset may not have captured the period of maximal PHE formation, which is known to have a more prolonged trajectory [5].

## Conclusions

In conclusion, this exploratory post hoc analysis of participants with ICH from the i-DEF randomized trial identified novel signals for an association of Hb with PHE, which warrant further exploration in future research investigating anemia as a potential treatment target for ICH.

## Supplementary Information

Below is the link to the electronic supplementary material.

Supplementary file1 (DOCX 175 kb)
