# Supplementary material for: Hemoglobin and Perihematomal Edema After Intracerebral Hemorrhage: A Post Hoc Analysis of the i-DEF Trial
Source: Neurocrit Care. 2025 May 21;44(1):64–71. doi: 10.1007/s12028-025-02284-3 (PMC12819462; doi:10.1007/s12028-025-02284-3)
Supplement: Supplementary file 1 — Supplementary file1 (DOCX 175 kb) [file 12028_2025_2284_MOESM1_ESM.docx]

**Supplementary Material**

Hemoglobin and perihematomal edema after intracerebral hemorrhage: a post-hoc analysis of the i-DEF trial

Polymeris AA, Lioutas VA, Marchina S, Seiffge DJ, Roh DJ, Poyraz FC, Selim MH, i-DEF Investigators

**Supplementary Figure 1.** Workflow of the assessment of perihematomal edema


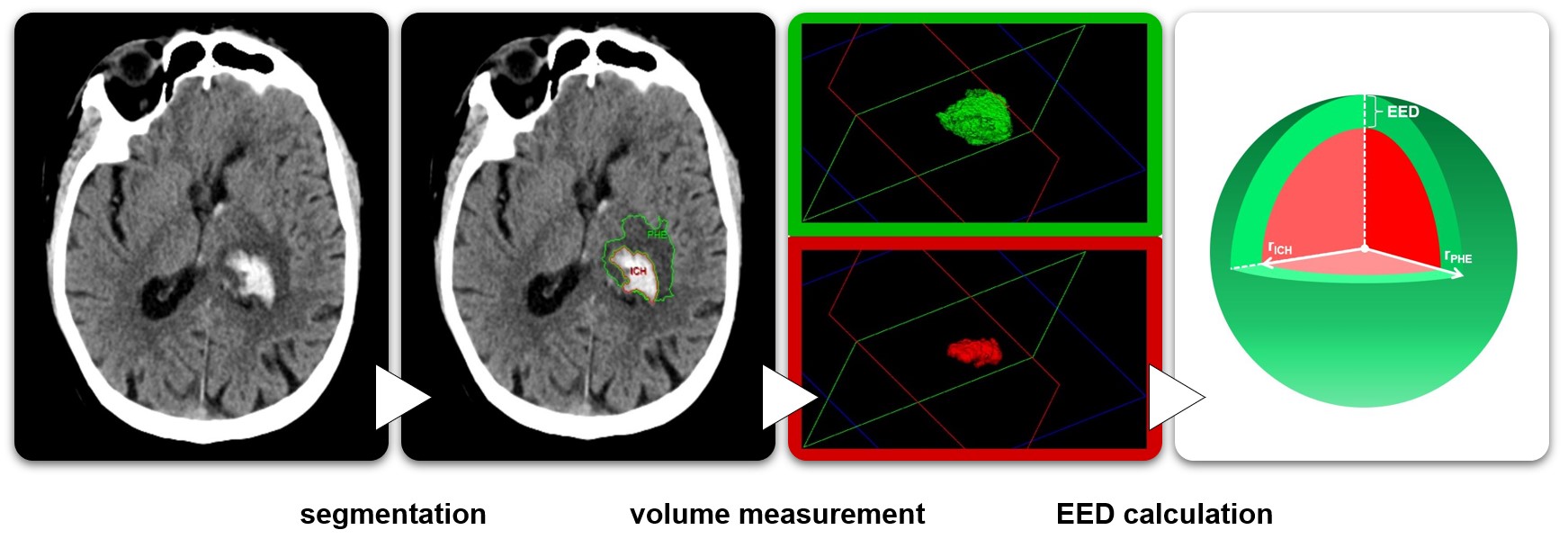


- Segmentation of intracerebral hematoma (ICH; red) and perihematomal edema (PHE; green) is done using a semi-automated approach based on density thresholds with subsequent manual correction on each slice using the ‘Analyze’ imaging analysis software.
- The software outputs the volume of ICH and PHE using the segmentation data.
- The volume data of each participant are used to calculate further PHE metrics. Relative PHE is calculated by dividing the PHE volume by the ICH volume. Edema extension distance (EED) is calculated as the difference between the radius of a sphere equal to the combined ICH and PHE volume and the radius of a sphere equal to the ICH volume alone, according to the following formula (Parry-Jones et al, Stroke 2015):

EED = r_PHE_ – r_ICH_, where r_PHE_ = $\sqrt[3]{\frac{ICH volume+PHE volume}{\frac{4}{3}\pi}}$, and r_ICH_ = $\sqrt[3]{\frac{ICH volume}{\frac{4}{3}\pi}}$
